# Assessing system-based trainings for primary care teams and quality-of-life of patients with multimorbidity in Thailand: patient and provider surveys

**DOI:** 10.1186/s12875-019-0951-6

**Published:** 2019-06-17

**Authors:** Paibul Suriyawongpaisal, Wichai Aekplakorn, Borwornsom Leerapan, Fatim Lakha, Samrit Srithamrongsawat, Suparpit von Bormann

**Affiliations:** 1 Department of Community Medicine, Faculty of Medicine Ramathibodi Hospital, Mahidol University, Bangkok, Thailand; 2 NHS Lothian, Edinburgh, Scotland, UK; 3 The University of Edinburgh, Edinburgh, Scotland, UK; 4 Boromarajonani College of Nursing, Nonthaburi, Thailand

**Keywords:** Capacity building, Human resource development, Primary care team (PCT), Health-related quality of life (HRQoL), Health systems strengthening, District health systems (DHS), Thailand, Low- and middle-income countries (LMICs), Multilevel modelling, Generalized linear mixed model (GLMM)

## Abstract

**Background:**

Strengthening primary care is considered a global strategy to address non-communicable diseases and their comorbidity. However, empirical evidence of the longer-term benefits of capacity building programmes for primary care teams contextualised for low- and middle-income countries is scanty. In Thailand, a series of system-based capacity building programmes for primary care teams have been implemented for a decade. An analysis of the relationship between these systems-based trainings in diverse settings of primary care and quantified patient outcomes was needed.

**Methods:**

Facility-based and community-based cross-sectional surveys were used to obtain data on exposure of primary care team members to 11 existing training programmes in Thailand, and health profiles and health-related quality of life of their patients measured in EuroQol-5 Dimension (EQ-5D) scale. Using a multilevel modelling, the associations between primary care provider’s training and patient’s EQ-5D score were estimated by a generalized linear mixed model (GLMM).

**Results:**

While exposure to training programmes varied among primary care teams nationwide, District Health Management Learning (DHML) and Contracting Unit of Primary Care (CUP) Leadership Training Programmes, which put more emphasis on bundling of competencies and contextualising of applying such competencies, were positively associated with better health-related quality of life of their multimorbid patients.

**Conclusions:**

Our report provides systematic feedback to a decade-long investment on system-based capacity building for primary care teams in Thailand, and can be considered as new evidence on the value of human resource development in primary care systems in low- and middle-income countries. Building multiple competencies helps members of primary care teams collaboratively manage district health systems and address complex health problems in different local contexts. Coupling contextualised training with ongoing programme implementation could be a key entity to the sustainable development of primary care teams in low and middle income countries which can then be a leverage for improving patients outcomes.

**Electronic supplementary material:**

The online version of this article (10.1186/s12875-019-0951-6) contains supplementary material, which is available to authorized users.

## Background

Multimorbidity, the co-existence of more than one chronic condition in one person, has increasingly posed a major challenge to existing models of healthcare delivery. In fact, multimorbidity in low- and middle-income countries (LMICs) is more complex than that of high-income countries as it usually combines non-communicable diseases (NCDs), infectious chronic diseases, and injuries [1]. To cope with such difficulties, the current healthcare delivery models in LMICs need to be shifted towards primary care models that can provide first contact and continuous, coordinated, and comprehensive care for individuals and families [2].

Literature also suggests human resource development for primary care in LMICs should be contextualised to their settings, and that primary care practice should be redesigned to tackle the challenge of NCDs in resource-limited countries by focusing more on patients and communities, integration of services, innovative service delivery, and adoption of new technologies for communication [3, 4]. Yet many agencies have paid substantial attention to strengthening primary care in LMICs they tend to have focused only on traditional health threats, such as infectious diseases and maternal and child health [3].

In Thailand, primary health care has long been a focus of national healthcare reforms [5], and has gradually transformed towards a more comprehensive, integrated, people-centred health services [6, 7]. The country is recognised as a ‘trailblazer’ amongst LMICs in achieving successful implementation of universal health coverage (UHC) [8, 9]. Thailand’s UHC is not considered the ‘big bang’ reform, but rather a result of successive governmental investments on financial risk protection and improving coverage of health services via a series of incremental changes since 1970s—spearheading by continuous development of health care infrastructure and workforce [10]. Currently, more than 98% of the Thai population are covered by one of the three financial risk protection schemes: the Civil Servant Medical Benefit Scheme (CSMBS) provides cover for civil servants and their dependents (9%); the Social Health Insurance (SHI) provides cover for private sector employees (15%), and the Universal Coverage Scheme (UCS) provides cover for those not enrolled in either CSMBS or SHI schemes (75%) [11]. Among the three schemes, the UCS put the greatest emphasis on primary care development, as its primary care networks were given greater control of financial resources to developing community-based health facilities that provide integrated medical and public health services and its beneficiaries are entitled to free outpatient and inpatient services at their registered state-run health facilities within local networks of District Health System (DHS) [12].

Thailand is self-reliant as regards its healthcare workforce production and recognises the gaps in workforce density especially within the primary care workforce. Thailand has a limited number of physicians per population with approximately 36,000 physicians in the total workforce serving a population of over 62 million [13], thus the provision of primary care in most DHSs of Thailand has relied heavily on a non-physician workforce like many other LMICs [14]. To combat these gaps Thailand has chosen to adopt a “task shifting” strategy as a long-term approach to training health workers to work in primary care settings [13]. However, instead of using oversea resource as often happens in many LMICs Thailand’s capacity building programmes for primary care teams (PCT) were implemented by local experts and local financing mechanisms [12, 15]. Since 2006, a series of 11 training programmes for multidisciplinary PCTs have been implemented nationwide, aiming to prepare the workforce to cope, both with the rapidly growing demand for healthcare due to increased access post implementation of the UHC policy in 2002, and to manage the country’s epidemiological transition from infectious to chronic non-communicable disease (NCDs) and an aging population [15] (Table 5 in Appendix).

**Fig. 1:**
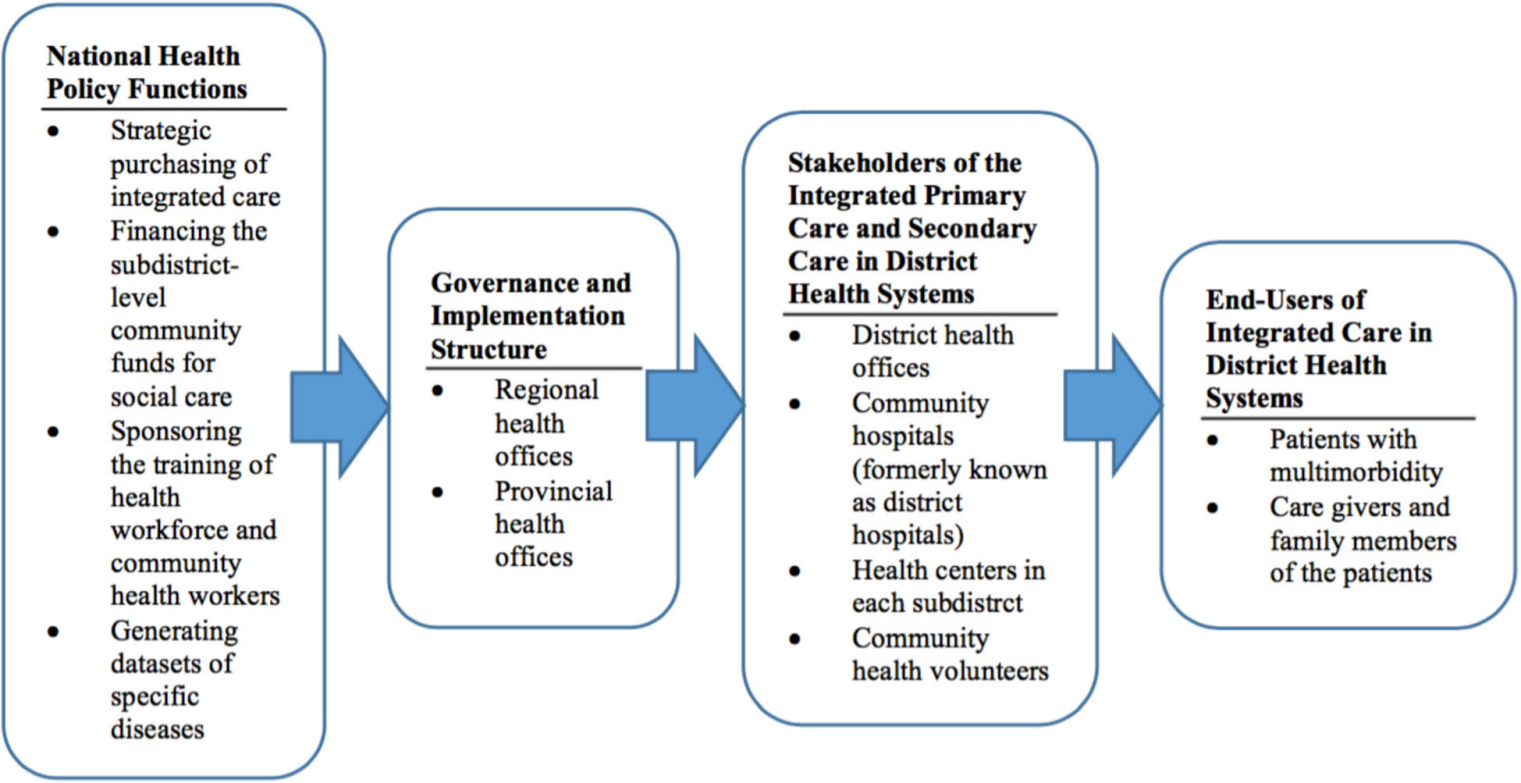
Structures and functions for the provision of integrated primary care under the Universal Coverage Scheme (USC) at the district level of Thailand

The direction of these system-based training programs was unified towards building knowledge and skills in providing integrated care at the district level for all UCS beneficiaries, under the governance of the National Health Security Office (NHSO) as shown on Fig. 1 although the contents and scope varied.

Several training programmes, namely “District Health Management Learning (DHML)” and “Contracting Unit of Primary Care (CUP) Leadership Training”, focused on bundling of competencies for managing integrated primary care systems, including resource sharing, community participation and inter-sectoral collaboration, health information systems, management skills of the leaders, coordination and unity of teamwork, and integrated service delivery. Some programmes, such as the “Family Practice Learning (FPL)” and the “District Health Systems (DHS) Appreciation Training” had a narrower scope focusing primarily on managing ambulatory care in the district hospitals or on self-assessment of PCTs respectively. Other programmes were based on community health programmes, but not directly related to skills for primary care practices. For all programme mobilization of community health volunteers as well as local funds were considered a major focus.

Similar to primary care in other LMICs, there has been limited systematic assessment of the effects of these capacity building programmes on health outcomes both in Thailand and in other LMICs. A systematic review of mental health training for primary care workers and community health workers in Africa showed that most studies had methodological limitations, particularly with respect to the absence of controlled evaluations and use of short-term assessments and most focused on knowledge and attitude, with only one assessing patient outcomes [16]. Given that strategies of each training programmes are heterogeneous, empirical analyses that can reveal the impacts of these contextualised training programmes in diverse primary care settings of Thailand on health outcomes of multimorbid patients are needed. We investigated the relationship between exposure of PCTs to systems-based capacity building programmes in Thailand and health-related quality of life of their patients with multimorbidity.

## Methods

The present study employed a multilevel, cross-sectional analysis of individual patients with multimorbidity and of the PCT trainings, clustered in the districts of eight selected provinces in three geographical regions of Thailand. Because the training programmes were embedded in multifaceted national policy interventions, patient outcomes might have been impacted by multiple factors, including selection bias and other changes due to history, maturation, secular trends or other endogenous change. Multilevel modelling enabled us to explore the relationships between the patient outcomes and the exposure to system-based training programmes of their primary care providers, and is considered an optimal approach to tease out the programme effects of such training programmes on the patient outcomes [17, 18].

Two parallel-hierarchical, cross-sectional surveys were conducted during October 2015—a patient survey enquiring about their health profiles and their experience of utilising primary care in local communities, and a survey of PCTs exposure to various training programmes and their work experience. Sample size was calculated for the patient level and the provider level, with statistical power dependent on the design effect and the total sample size for each level [19].

Sample size for PCT survey was calculated with sufficient number of participants to identify approximately 25% of PCT members previously exposed to system-based training programmes (*n* = 1.96^2^ x (P)(1-P)/d^2^ where d = 0.05), and the estimated sample size for PCT members was 288. Sample size for the patient survey was calculated to identify the multimorbid Thai patients with an average EQ-5D score of 65 (± S.D. of 46) [20]. The sample size calculation for patients took into the account of the design effect of 6.5 (*n* = (1.96^2^ x σ^2^/ (E^2^)) x DE, where σ =46, E = margin of error of 5, DE = design Effect as determined by (1+ (m-1) x ICC), where m = number of sites of 24, ICC = intraclass correlation coefficient of 0.25, and thus the estimated sample size for patient’s survey was 2113.

A multi-stage, clustered sampling was used to conduct our survey of patient’s health profiles and their experiences of utilising primary care. The three most populated regions of the country (northeast, central, south) were selected to participate in the study, excluding the regions which the top executives did not allow their PCTs to participate in our survey. Within each of these three regions, two to three provinces were chosen with the size of population of those provinces being proportional to size of the population in each region. In each province, the most urban district and two adjacent rural districts were selected, based on the number of training programmes that the PCTs in each district were exposed to (Table 6 in Appendix). As a result, 24 districts in eight provinces were selected.

Ethical approval was obtained prior to data collection from the Institutional Ethical Review Board of the Faculty of Medicine Ramathibodi Hospital, Mahidol University.

### Selection of patients

In accordance with UCS policy, the population in each sub-district are enrolled at local health centres, which serve as their primary care organisation [10]. In each health centre, patients with multimorbidity are categorised within the patient registry into four groups: the chronically ill, the elderly, the disabled, and the terminally ill. Although these four groups were not mutually-exclusive by nature, each patient was registered by only the most severe condition in terms of functional limitations. Hence, these four groups were administratively considered mutually-exclusive, enabled for selection via random sampling of multimorbid patients from each group.

Due to the small size within the category of the terminally ill, the patients who are in dying processes defined as the last minutes or days of life when death is obviously imminent [21], were used as index cases to start with. Then, the patients from other three groups who resided in the same community were selected by simple random sampling, with the number of samples in each group proportional to the size of the corresponding patient group in the community. The average number of sampled patients per each primary care organization were six terminally ill, eleven disabled, eight elderly persons with functional limitations, and 53 chronically ill patients.

### Selection of PCT team members

All the members of PCT health personnel of each team were invited to participate in the survey. The average number of members was 10 persons per team, with a range of eight to 12 persons per team, and the average number of members who participated was nine persons.

### Data from the patient survey

Data were obtained from patients by face-to-face interviews during household visits, using a standard set of questions for assessment of HRQoL. In addition, a cognitive assessment tool and a questionnaire assessing patient’s health profiles and experiences with utilising health services were also conducted.

The HRQoL questionnaire consisted of one page of the Thai version of the EuroQol-5 Dimensions (EQ-5D), a generic instrument widely used for describing health outcomes and already was validated in the Thai population [22, 23]. The five dimensions of impairment are mobility, self-care, usual daily activities, pain or discomfort, anxiety or depression. Each dimension is measured in 5-Likert scale from no impairment to extreme impairment. It took five to 7 minutes to administer the face-to-face test by a trained nurse (Additional file 1).

The Thai version of Rowland Universal Dementia Assessment Scale (RUDAS-Thai) [24] was used to predict the performance of patients on tests of verbal learning, executive function, working memory, visuospatial function, and motor function. We used this cognitive assessment tool to rule out patients with impaired cognitive functions that might compromise the validity and the reliability of their responses to our patient survey. It took eight to 10 minutes to administer the face-to-face test by a trained nurse. A cut-off point of 25 or less was used to maximize sensitivity of the screening test. The questions for patients, caregivers and stakeholders are shown on the Additional file 1.

### Data from the PCT survey

A self-administered questionnaire was distributed to all members of the PCTs in the selected community hospitals and health centres in the selected 24 districts. This self-administered standard questionnaire comprised exposure to training on specific topics and further training needs, as well as other items including team structure and functions, channels and frequency of communication among team members, experience of working within a PCT, and work experiences. A total of 11 training programmes were identified from reviewing governmental documents, and all included in the questionnaire. The questions for leaders and members of primary care team are shown on the Additional files 2 and 3.

### Data analysis

Due to the hierarchical structure of our data, a multilevel analysis was used to consider the patient data (level 1) nested within the PCT data (level 2). Districts were grouped into provinces in order to achieve the power of test and to reflect a more realistic contextual effect on the trainings at provincial level. The percentage of exposure by each type of PCT member was estimated. Variance of exposure to the programmes by each type of PCT member was also estimated. Multi-level modelling was used to distinguish between the effects of training-programme on PCTs and the effects of trained PCTs on patients. The association between EQ-5D score and selected predictor variables (morbidity, regularly visited healthcare facility, age, sex) were tested using a generalized linear mixed model (GLMM) [25].

The multilevel analysis of GLMM began with a two-level null (empty) model with no predictor variables in the fixed part, and only the intercepts in the random part of the model (M1). This model was used as a reference for comparing the size of contextual (province) variations in EQ-5D scores in subsequent models. The next model (M2) included patient-level variables as the fixed effects only. Finally, the explanatory variables at the patient level and provincial level were added as fixed effects resulting in the best model fit (M3).

The variance partition coefficients at each level were calculated using the restricted maximum likelihood. The random part results of the null model (M1) are reported together with the corresponding intra-class correlations at the district level. Variance partition coefficients with corresponding two-sided 95% confidence intervals (CI) are provided. The significance level was set to 5% (two-sided). All statistical analyses were carried out using Statistical Package for the Social Science for Windows (SPSS) version 18.0 (SPSS Inc., Chicago, IL, USA), with the exception of the multilevel analysis that was conducted by using the procedure “mixed” in Stata statistical software version 10 (Stata Corp, College Station TX, USA).

## Results

### The patient outcomes (level 1)

A total number of 1916 participated in our cross-sectional survey (88.9%). Approximately two thirds (62.8%) of patient samples self-completed the survey, whilst just over a third (37.2%) responded by their care givers as the proxy respondents due to cognitive impairment of the patients, as measured by our cognitive assessment tool. Mean age was 67.5 years and 60% were female. Almost 90% lived with multimorbidity and 63% were terminally ill. Table 1 details their health profiles and other attributes, including residential provinces and type of healthcare facilities they regularly visited.

**Table 1:** Patients’ health profiles and other attributes (*N* = 1874^a^)

Demographic and health profile	Percent
Age (years) (mean, SD)	67.5, 17.4
Sex (%)	
men	39.60
women	60.40
Patient group (%)	
elder/disabled/chronic disease/palliative	35.9
elder/disabled/palliative	10.3
elder/disabled/chronic diseases	1.7
elder/disabled	0.7
elder/chronic diseases	1.4
elder	4.0
disabled/chronic diseases/palliative	1.9
disabled/palliative	8.9
disabled	5.1
chronic diseases/palliative	2.9
chronic diseases	24.8
palliative	2.6
Residential province (% in population of all ages)	
northeast 1	19.20
northeast 2	8.20
central 1	19.80
central 2	12.80
central 3	10.10
south 1	17.90
south 2	7.00
south 3	5.00
Healthcare utilization: healthcare facilities regularly visited (%)	
referral hospitals	51.90
others hospitals	6.80
district hospitals	15.90
health centers	25.40

^a^excluding samples with missing data

Mean EQ-5D score of our patient samples was 69 (± S.D. of 42). To provide a global impression, the percentage of patient samples reporting HRQoL being compromised from moderate to severe degree, according to the EQ-5D measure, is summarized in Table 2. Mobility was the most reported dimension of diminished quality of life (44% of respondents).

**Table 2:** Percentage of patients reported quality of life being compromised from moderate to severe degree according to EQ-5D (*N* = 1874^a^)

Dimension	Percent
Mobility	44
Self-care	35
Usual activities	38
Pain or discomfort	21
Anxiety or depression	9

^a^excluding samples with missing data

As shown on Table 3, the association between the EQ-5D score and selected predictor variables (morbidity, age, sex, regularly visited healthcare facility) were tested by using GLMM. Although both single morbidity and multimorbidity were inversely associated with the QoL, only the association between single morbidity and the QoL was statistically significant (*p* = 0.033).

**Table 3:** Parameter estimates of GLMM (level 1) with EQ-5D scores as dependent variable adjusted for patients’ age, sex, and regularly visited healthcare facilities (*N* = 1874^c^)

Parameter Estimates	ß	Std. Error	95% Confidence Interval	
			Lower	Upper
(Intercept)	13.927	.7975	12.364	15.490
Single morbidity	−1.764	.8271	−3.385	−.143
Double morbidity	−1.554	.8245	−3.170	.062
Three morbidity	−1.585	.8716	−3.293	.124
Four morbidity	0^a^			
(Scale)	34.982^b^	1.1456	32.807	37.301

^a^Set to zero because this parameter is redundant

^b^Maximum likelihood estimate

^c^excluding samples with missing data

### The PCT outcomes (level 2)

A total of 218 PCT members responsible for patients living in the selected districts participated in our survey. Amongst all professions in the PCTs, the highest percent of participants were nurses (29.8%), physiotherapists (16.5%) and community health volunteers (16.5%). The average proportion of PCT members reportedly exposed to each system-based training programmes was 26.3% among all PCT members. The majority of PCTs (88%) in the selected districts were exposed to less than five training programmes from the total of 12 programmes. Percentage of exposure to the selected training programmes among PCT members varied, with the most limited exposure (6.42%) being to the training on CUP Leadership and the most frequent exposure (76.15%) being to the training on DHML. The distribution of PCT exposure to selected training programmes shown in detail on Table 7 in Appendix.

Among 11 identified training programmes, only four were included in the final regression model: DHML, DHS Appreciation, CUP Leadership, and FPL. Programmes with low uptake by PCT members or deemed by the research team to have content less relevant to capacity building were excluded. Results of multilevel analysis on PCT, detailed on Table 4, show that HRQoL were associated to the PCT exposure to three training programmes: DHML, CUP Leadership and FLP. However, associations between the three training programmes and patient’s HRQoL were not consistently positive, as FPL was negatively associated with patient’s HRQoL. Additionally, the duration of time spent working within the PCT was also slightly negatively associated with EQ-5D score of their patients.

**Table 4:** Parameter estimates of the final GLMM (the multilevel model) with EQ-5D score as dependent variable (*N* = 1865^a^)

Parameters	Values of parameters				
Score	Coef.^b^	Std. Err.	z	95% Conf. Interval	
				Lower	Upper
*Level 1 (the individual patient level)*					
Sex	−0.49	0.28	−1.78	−1.04	0.05
Age	0.01	0.01	1.12	−0.01	0.02
*Level 2 (the PCT level)*					
Intercept	17.69	2.45	7.21	12.89	22.50
Months spent within PCTs	−0.02	0.00	−3.69	−0.04	− 0.01
District Health Management Learning (DHML) Programme	8.61	2.49	3.45	3.73	1.53
Contracting Unit of Primary Care (CUP) Leadership Training Programme	17.43	5.53	3.15	6.58	22.50
District Health Systems (DHS) Appreciation Training Programme	−3.86	2.75	−1.40	−9.25	−8.46
Family Practice Learning (FPL) Programme	−16.64	4.18	−3.99	−24.83	−28.27

^a^excluding samples with missing data

^b^Variance partition coefficients

## Discussion

Our study provides an assessment of training programmes for PCTs that are closely-linked with domestic funding and national policies—with all programmes being embedded in multifaceted national policy interventions and implemented in overlapping periods during the past decade. Amid the deficiency of systematic, quantitative assessment of these training programmes, this report provides preliminary findings on how contextualized training programmes potentially impact the performance of PCTs taking care of patients with multimorbidity. This report provides new evidence on mobilising system-based approaches to human resource development of primary care systems in LMICs. Policy interventions supporting better trained PCTs must be prioritised, as they are vital to sustainable development goals and UHC in LMICs [26].

Our findings demonstrated that patient outcomes were positively associated with PCT exposure to training with a focus on bundling of competencies and contextualization—the DHML Programme addressed how PCTs should be continuously learning and providing relevant health services to their patients under the dynamic primary care contexts, and the CUP Leadership Programme enhanced the conceptual and managerial skills of hospital directors, who controlled healthcare resources of each DHS according to the purchasing model of UCS [10], in development of area-based primary care delivery systems. These competencies are consistent with existing evidence of effective “systems interventions” following the concepts of Wagner’s Chronic Care Model (CCM) [27–29].

In contrast, we found that programmes which demonstrated no relationship with patient outcomes had a narrow scope, as in the case of the FPL Programme. The relatively isolated training in the hospital settings of the FPL did not address the training needs of public health workers who were delegated to take care of patients with chronic conditions at health centres—a primary care setting that serves a significant share of patients (25.4%) of DHSs. In the case of the DHS Appreciation Programme, there was insufficient power to assess fully due to low coverage as where only 48/928 districts (5.2%) had access and it was only available for 1 year.

A slightly negative association between the duration of each health worker working in PCTs and their patient’s HRQoL was rather surprising. One possible explanation could be the high workload and the lack of health workforce management staff leading to health care personnel experiencing burnout, increased levels of stress or work disengagement, which in turn could be linked to undesirable patient outcomes [10]. Further studies are needed to explore if this explanation is valid, and which policies could have prevented this phenomenon.

As our findings were drawn from more than one capacity building programmes being implemented in overlapping periods over years, a timely assessment could have helped policymakers reallocating resources across programmes more effectively. Further investigation is needed for a better understanding of why each programme demonstrated different relationship with patient outcomes. Moreover, if more systematic, long-term impact monitoring and evaluation were put in place, it can help to start conversations and raise more questions of what the priorities are in Thailand’s primary care development, and which then need to be researched furthermore.

Lessons learned from these capacity building programmes for PCTs in Thailand can be applied in other LMICs, too. For instance, in African countries where developing sustainable family medicine training programmes is found to be feasible but slow to progress due to many obstacles such as lack of role models [30], adequate support from key figures at policy level and academic institutes for system-based capacity build is warranted. Using Thailand as example, finding sustainable sources of financing for such training programmes should be a major concern, as sustainability of primary care development is at risk when the countries rely only on partnerships, international financial supports and expertise from overseas.

This empirical study also contributes to a growing body of the literature that suggests human resource development for primary care in LMICs should be contextualised to their resource-limited settings. Communities are the critical entry points for prevention and control of NCDs, and experimental studies have shown that community leaders, civil society organizations, churches, and women’s groups are well positioned to raise awareness of NCDs and the importance of healthy lifestyles [31]. In Brazil, primary care encompasses health services delivered by physicians and community health workers, and task shifting from physicians to other health workers in a country with physician shortage is associated with good results at a low cost of treatments for complex patients, such as patients living with HIV/AIDS [2]. A longitudinal data analysis also demonstrated this model of primary care significantly contributed to mortality reduction from heart and cerebrovascular diseases [32].

Positive findings from our study in Thailand are consistent with a recent meta-analysis of 36 studies from developed countries, which revealed a possibly increased effectiveness of case management delivered by multidisciplinary primary care teams (PCTs) that including social workers for elderly with multimorbidity [33]. Capacity building of such high-performing PCTs has been highlighted by multiple components of specific team goals with measurable outcomes, detailed clinical and administrative systems, clear division of labour, a permanent training environment, and agreed-upon modes of communication within the team [34]. As evident by systematic reviews of chronic disease management in primary care settings, multi-component interventions including team-based training seem to be more effective than either standalone primary care interventions or standalone training of PCTs [35–37].

Limitations of this study include that although using EQ-5D score as the standardized measurement for patient outcomes make sense in our analysis of multimorbid patients whose HRQoL usually decreased at all level of comorbidity, previous studies indicate that the cumulative effect of chronic health conditions is not simply incremental—the consequences of specific disease combinations may have a greater effect on functional status, quality of life and mortality than others [22, 38]. With a limited sample size for patients who had more than one morbidity, we were not able to demonstrate the gradient of association as expected, and therefore further studies are needed.

## Conclusion and recommendations

Our report provides new evidence on a long-term approach to system-based trainings of PCTs amidst the rising challenges of aging, NCDs and associated multimorbidity in resource-limited settings of LMICs. The strengths of our study include applying a generalized linear mixed model to assess a multilevel association of the predictor variables and the standardized measure of patient’s quality of life. The multilevel analysis revealed a positive relationship between system-based trainings and patient outcomes in diverse settings of Thailand’s primary care for programmes with an emphasis on bundling of competencies for managing complex health problems in different local contexts are associated with desirable patient outcomes.

Our findings should be considered preliminary given the limitations of cross-sectional design to demonstrate causal relationship, limited sample size for specific groups of patients with more than one morbidity. Non-random sample selection may compromise external validity of the findings, while cross-sectional design of our study is inadequate to prove a causal relationship as other confounders such as disease duration or socio-economic status of the patients are not included. Despite a high response rate of our patient survey, potential non-response bias still can be made. The limitations of sample size given the multiple analyses should also be considered. Further studies would be needed to fill the gaps, for instance, by using a cohort study that takes into the account of multilevel factors.

Nonetheless, our study can be considered a “proof of concept” that the effective capacity building programmes for primary care in LMICs are beyond developing only clinical skills, and that trainings in isolation of programme implementation is not enough. It can help policymakers looking in a greater depth of monitoring and evaluation of existing capacity building programmes for PCTs and considering removing resources from programmes that show no positive relationship with patients’ health outcomes, and thus freeing up resources to spend on more functioning ones. Coupling contextualized training by bundling of competencies with an ongoing programme implementation and evaluation may be the key to the sustainable development of primary care providers in developing countries.

### Additional files

Additional file 1: Questions for Patients, Caregivers and Stakeholders. (DOCX 48 kb)

Additional file 2: Semi-structured Interview Guide for the Leaders of Family Care Team. (DOCX 26 kb)

Additional file 3: Questions for the Members of Family Care Team. (DOCX 2397 kb)

## Appendix

**Table 5:** List of training programs relevant to primary care

Project title (year of implementation)	Trainees	Objectives	Available implementation details	Assessment	Results: number of the targets completed the training and other outcomes
1. CUP leadership (2009–2013)	Managers of contracting unit for primary care (CUP): -district hospital directors or representative (physicians or dentists) -district health officers	To enhance conceptual and managerial skills in development of primary care suitable to area-based context and health needs	Classroom sessions: didactic lectures, group discussion based on experiences from management practices	- not clear	213 trainees from 200 districts
2. Training of family practice doctor (2006–2007)	Doctors from district hospitals and/or health centers	To enhance: -FMP in district hospitals and/or health centers -knowledge and skills in: applied psycho-analysis and patient communication; management of primary care network of practitioners	Classroom sessions: didactic lectures, group discussion based on experiences from clinical clerkship	Cognitive knowledge assessment before and after the training	200 trainees
3. In service training for family medicine practice (FMP) (2009–2015)	Second or third year medical graduates	To enhance FMP in district hospitals	-Week-end classroom sessions for 3 years: didactic lectures, group discussion based on experiences from real life practices −26 medical school faculties and affiliates as trainers	-Self assessment -Year-end summative assessment	91 trainees
4. Family practice learning (FPL)(2012–2014)	A multidisciplinary team of 3 to 5 members with at least 1 doctor or pharmacist in each team	To enhance team-based FMP in district hospitals and health centers	-classroom sessions for 1 year: didactic lectures, clinical rotation and community practice (home visits and community dialogues), case conferences	-Minimum requirement: a team report of family assessment and interventions -Individual portfolios of doctors or pharmacists -Comments of academic advisors	− 210 doctors −14 pharmacists − 1 dentist - over 1000 other health alliances such as nurses, public health workers, physiotherapists
5. District Health Management Learning (DHML) (2014- present)	A multidisciplinary team of 5 to 8 members from each district (district health officers, district hospital staffs (the directors and some of the followings: physicians, pharmacists, senior nurses or dentists), local authority, community leaders (village heads, sub-district heads, district head officers), organized groups of people (elderly clubs, housewife clubs))	To strengthen: resource sharing, unity in teamwork, community participation, health information systems, management skills of the leaders, coordination, integrated service delivery, inter-sectoral collaboration	class-based learning, clinical practice and community practice, standard practice guideline, team contest sessions (to encourage sharing of knowledge and practices)	-Individual self- assessment -Improvement of collaboration and coordination among key actors in DHS in terms of regularity, continuity and knowledge sharing	− 224 teams −44 emerging coordinating centers to perpetuate training of DHML in district health offices, district hospitals and academic institutes
6. District Health System (DHS) Appreciation (2011)	The same as no. 5	To consolidate lessons learned from implementation of DHS	Group sessions focusing on functions of multi-sectoral multidisciplinary collaboration and coordination towards innovations for delivery of essential care on continual basis with multi-source resource mobilization and development of health information systems	Self-assessment using broad thematic guideline: unity of the team, community participation, appreciation, resource sharing, essential care	48 districts
7. Community nurse training (2008–2011)	local high school graduates recruited by district hospital directors and senior nurses using verbal interview and results of local resident opinion survey	To produce graduate nurses with emphasis on community practices in order to enhance retention in district hospitals	- Nationally approved standard curricula for a nursing school (4-year period) - On-top clinical clerkship rotations in the summer, annually, at district hospitals where the nurse graduates will work during compulsory period of 8 years	-Collaborative recruitment and sponsorship by district hospitals, local administrative authorities and schools of nursing -Close monitoring by supervisor (a nurse and/or physician) from district hospitals during the training - national license examination	− 808 nurse graduates from 442 districts
8. General practice nurse training (2006–2007)	Graduate nurses from district hospitals or health centers	To enhance knowledge and skills in family practice	-clinical clerkship rotation under supervision of physicians -classroom sessions: didactic lectures, group discussion based on experiences from the clinical clerkship	Not clear	1000 nurses
9. Family and Community Pharmacist Learning (FCPL)(2014–2015)	Pharmacists from district hospitals	To enhance knowledge and skills in family practice	-classroom sessions: didactic lectures, group discussion based on experiences from clinical clerkship	-A report on drug delivery models for home-based and community-based settings -A report of case studies of continuity of care from home to hospital	59 trainees
10. Supervisors in primary care practice (2006–2007)	Supervisors from district hospitals and district health offices	To enhance knowledge and skills in human resource development	Not clear	Cognitive knowledge assessment before and after the training	4100 trainees
11. Training of clinical health workers in primary care (2007)	Health workers in health centers or primary care unit in hospitals	To enhance knowledge and skills in primary care practice	Not clear	Cognitive knowledge assessment before and after the training	18,000 trainees
12. Training of public health workers in primary care (2006–2007; 2015)	public health workers in health centers	To enhance knowledge skills and attitude in public health functions: community diagnosis, project planning and implementation	Classroom activities: case studies, didactic lectures, group discussion	Cognitive knowledge assessment before and after the training	720 trainees

**Table 6:** Number of individual patient samples (N) distributed by district with the number of previous exposure to training programs

Province in each region	district	Number of exposure to training programs	N
Central 1	urban	5	140
	rural	2	48
	rural	5	52
Central 2	urban	4	200
	rural	2	40
	rural	5	120
Central 3	urban	1	64
	rural	3	52
	rural	5	60
Northeast 1	urban	1	28
	rural	4	32
	rural	4	80
Northeast 2	urban	1	40
	rural	2	160
	rural	4	140
South 1	urban	3	240
	rural	2	32
	rural	4	16
South 2	urban	2	88
	rural	2	12
	rural	4	80
South 3	urban	1	56
	rural	4	16
	rural	2	120
Total			1916

**Table 7:** Percentage of each type of members in primary care team exposed to selected training programs included in the final multilevel model (*N* = 218)

Profession/Position of PCT members	Exposure to Selected Training programmes (%)			
	District Health Management Learning (DHML)	District Health Systems (DHS) Appreciation Training	Contracting Unit of Primary Care (CUP) Leadership Training	Family Practice Learning (FPL)
Physician (*n* = 12)	75	8.33	0	8.33
Dentist (*n* = 1)	100	0	0	0
Pharmacist (*n* = 8)	87.50	50	0	37.50
Nurse (*n* = 65)	83.08	23.08	7.69	15.38
Physiotherapist (*n* = 15)	73.33	26.67	0	20
Public health worker (*n* = 36)	75	16.67	8.33	8.33
Dental assistant (*n* = 3)	33.33	0	0	33.33
Traditional medicine worker (*n* = 7)	71.43	42.86	0	28.57
Nutritionist (*n* = 3)	100	33.33	0	0
Other health personnel (*n* = 12)	58.33	8.33	8.33	0
Community health volunteer (*n* = 36)	72.22	16.67	8.33	16.67
Local authority officer (*n* = 5)	80	40	20	20
Sub-district head (*n* = 1)	100	0	0	0
Village head (*n* = 6)	66.67	33.33	16.67	33.33
Other volunteers (*n* = 2)	50	50	0	50
Others (*n* = 6)	83.33	16.67	0	16.67
Average exposure of all PCT members (*n* = 218)	76.15	21.56	6.42	15.6

## Abbreviations

CCM: Chronic Care Model
CI: Confidence interval
CUP: Contracting unit for primary care
DHML: District Health Management Learning
DHS: District Heath System
EQ-5D: EuroQol-5 Dimensions
FPL: Family practice leadership
GLMM: Generalized linear mixed model
HIV/AIDS: Human immunodeficiency virus/acquired immune deficiency syndrome
HRQoL: Health-related quality of life
LMIC: Low- and middle-income country
NCD: Non-communicable disease
NHSO: National Health Security Office
PCT: Primary care team
RUDA: Rowland Universal Dementia Assessment Scale
SD: Standard deviation
UHC: Universal health coverage

## References

[1] Oni T, McGrath N, BeLue R, Roderick P, Colagiuri S, May CR (2014). Chronic diseases and multi-morbidity - a conceptual modification to the WHO ICCC model for countries in health transition. BMC Public Health.

[2] Starfield B, Shi L, Macinko J (2005). Contribution of primary care to health systems and health. Milbank Q.

[3] Kruk ME, Nigenda G, Knaul FM (2015). Redesigning primary care to tackle the global epidemic of noncommunicable disease. Am J Public Health.

[4] World Health Organization (2007). Everybody business - strengthening health systems to improve health outcomes: WHO’s framework for action. The WHO document production services.

[5] Nitayarumphong S (1990). Evolution of primary health care in Thailand: what policies worked?. Health Policy Plan.

[6] Valentijn PP, Schepman SM, Opheij W, Bruijnzeels MA (2013). Understanding integrated care: a comprehensive conceptual framework based on the integrative functions of primary care. Int J Integr Care.

[7] Toro N. Who global strategy on integrated people-centred health services (IPCHS) / Estrategia mundial en servicios de salud integrada centrado en las personas (IPCHS). Int J Integr Care. 2015:15(8); WCIC Conf Suppl; URN:NBN:NL:UI:10-1-117366.

[8] Tangcharoensathien V, Witthayapipopsakul W, Panichkriangkrai W, Patcharanarumol W, Mills A (2018). Health systems development in Thailand: a solid platform for successful implementation of universal health coverage. Lancet.

[9] Rieger M, Wagner N, Bedi AS (2017). Universal health coverage at the macro level: synthetic control evidence from Thailand. Soc Sci Med.

[10] Evans TG, Chowdhury AMR, Evans DB, et al. Thailand’s Universal Coverage Scheme: Achievements and Challenges. An indepedent assessment of the first 10 years (2001-2010). Nonthaburi: Health Insurance System Research Office; 2012.

[11] Tangcharoensathien V, Limwattananon S, Patcharanarumol W, Thammatacharee J, Jongudomsuk P, Sirilak S (2015). Achieving universal health coverage goals in Thailand: the vital role of strategic purchasing. Health Policy Plan.

[12] Tangcharoensathien V, Pitayarangsarit S, Patcharanarumol W, Prakongsai P, Sumalee H, Tosanguan J (2013). Promoting universal financial protection: how the Thai universal coverage scheme was designed to ensure equity. Health Res Policy Syst.

[13] Wibulpolprasert S, Sirilak S, Ekachampaka P, Wattanamano N (2011). Thailand Health Profile 2008-2010. The war veterans Organization of Thailand Press, editor. Thailand Health Profile.

[14] Campbell J, Buchan J, Cometto G, David B, Dussault G, Fogstad H (2013). Human resources for health and universal health coverage: fostering equity and effective coverage. Bull World Health Organ.

[15] National Health Security Office (2010). The development of patient care and chronic disease with high costs.

[16] Liu G, Jack H, Piette A, Mangezi W, Machando D, Rwafa C (2016). Mental health training for health workers in Africa: a systematic review. Lancet Psychiatry.

[17] Stawski RS (2013). Multilevel Analysis: An Introduction to Basic and Advanced Multilevel Modeling (2nd Edition). Struct Equ Model Multidiscip J.

[18] Songkhla MN (2009). Health before profits? Learning from Thailand's experience. Lancet.

[19] TAB S (2005). Power and Sample Size in Multilevel Linear Models.

[20] Sakthong P, Kasemsup V (2012). Health utility measured with EQ-5D in Thai patients undergoing peritoneal dialysis. Value Health.

[21] Wilson DM, Woytowich B (2014). What proportion of terminally ill and dying people require specialist palliative care services?. Int J Palliat Care.

[22] Bayliss EA, Bayliss MS, Ware JE, Steiner JF (2004). Predicting declines in physical function in persons with multiple chronic medical conditions: what we can learn from the medical problem list. Health Qual Life Outcomes.

[23] Pattanaphesaj J, Thavorncharoensap M, Ramos-Goñi JM, Tongsiri S, Ingsrisawang L, Teerawattananon Y (2018). The EQ-5D-5L valuation study in Thailand. Expert Rev Pharmacoecon Outcomes Res.

[24] Limpawattana P, Tiamkao S, Sawanyawisuth K, Thinkhamrop B (2012). Can Rowland universal dementia assessment scale (RUDAS) replace mini-mental state examination (MMSE) for dementia screening in a Thai geriatric outpatient setting?. Am J Alzheimers Dis Other Dement.

[25] Breslow NE, Clayton DG (1993). Approximate inference in generalized linear mixed models. J Am Stat Assoc.

[26] Fairall L, Bateman E (2017). Health workers are vital to sustainable development goals and universal health coverage. BMJ.

[27] Bodenheimer T, Wagner EH, Grumbach K (2002). Improving primary Care for Patients with Chronic Illness. JAMA.

[28] Wagner EH, Coleman K, Reid RJ, Phillips K, Abrams MK, Sugarman JR (2012). The changes involved in patient-centered medical home transformation. Prim Care.

[29] Boyd CM, Boult C, Shadmi E, Leff B, Brager R, Dunbar L (2007). Guided Care for Multimorbid Older Adults: Kathleen Walsh Piercy, PhD, Editor. Gerontologist.

[30] Flinkenflögel M, Essuman A, Chege P, Ayankogbe O, De Maeseneer J (2014). Family medicine training in sub-Saharan Africa: south–south cooperation in the Primafamed project as strategy for development. Fam Pract.

[31] Fu D, Fu H, McGowan P, Shen Y-E, Zhu L, Yang H (2003). Implementation and quantitative evaluation of chronic disease self-management programme in Shanghai, China: randomized controlled trial. Bull World Health Organ.

[32] Rasella D, Harhay MO, Pamponet ML, Aquino R, Barreto ML (2014). Impact of primary health care on mortality from heart and cerebrovascular diseases in Brazil: a nationwide analysis of longitudinal data. BMJ.

[33] Stokes J, Panagioti M, Alam R, Checkland K, Cheraghi-Sohi S, Bower P (2015). Effectiveness of case management for “at risk” patients in primary care: a systematic review and meta-analysis. Quinn TJ, editor. PLoS One.

[34] Grumbach K, Bodenheimer T (2004). Can health care teams improve primary care practice?. JAMA.

[35] Vöhringer PA, Castro A, Martínez P, Tala Á, Medina S, Rojas G (2016). Healthcare team training programs aimed at improving depression management in primary care: a systematic review. J Affect Disord.

[36] Mitchell GK, Burridge L, Zhang J, Donald M, Scott IA, Dart J (2015). Systematic review of integrated models of health care delivered at the primary–secondary interface: how effective is it and what determines effectiveness?. Aust J Prim Health.

[37] Renders CM, Valk GD, Griffin SJ, Wagner EH, Eijk van JT, Assendelft WJJ (2001). Interventions to improve the Management of Diabetes in primary care, outpatient, and community settings: a systematic review. Diabetes Care.

[38] Gijsen R, Hoeymans N, Schellevis FG, Ruwaard D, Satariano WA, van den Bos GA (2001). Causes and consequences of comorbidity: a review. J Clin Epidemiol.

