# Supplementary material for: Assessing system-based trainings for primary care teams and quality-of-life of patients with multimorbidity in Thailand: patient and provider surveys
Source: BMC Fam Pract. 2019 Jun 17;20:85. doi: 10.1186/s12875-019-0951-6 (PMC6580542; doi:10.1186/s12875-019-0951-6)
Supplement: Supplementary file 1 — Questions for Patients, Caregivers and Stakeholders. (DOCX 48 kb) [file 12875_2019_951_MOESM1_ESM.docx]

**Additional file 3**

**Questions for Patients, Caregivers and Stakeholders**

| Variable | Sequence | | | Region | | | | | Province | | | | District | | |
| --- | --- | --- | --- | --- | --- | --- | --- | --- | --- | --- | --- | --- | --- | --- | --- |
| CODE 1 - 4 |  | | |  | | | | |  | | | |  | | |
| **Module 1 Nature of interviewee** | | | | | | | | | | | | | CODE | | |
| 1. Status | | | | | | | | | | | | | 5 | | |
| ☐1 self-report | | ☐2 Answer via unpaid caregiver caregiver/family members | | | | | | ☐3 Answer via paid caregiver | | | | ☐4 Neighbor/other....... |  |  |  |
| 2. Type (Can select more than one) | | | | | | | | | | | | | 6 - 9 | | |
| - - - 2.1 Elderly     - 2.2 Diabled     - 2.3 Patients with NCDs: specifiy………………………………………     - 2.4 Patients with palliative care | | | | | | | | | | | | |  |  |  |
| 3. Gende | | | - - - 1 male | | | | | | | ☐ 2 female | |  | 10 | | |
| 4. Age | | | …………………yr | | | | | | | | | | 11 | | |
| 5. Total family member....................persons | | | | | | | | | | | | | 12 | | |
| 6. Income of caregivers? | | | | | ☐1 No | | | | | | ☐2 Yes | | 13 | | |
| 7. Health benefits (Can select more than one) | | | | | | | | | | | | |  | | |
| - 1. UCS - 2. SSS - 3. Commercial health insurance from employer - 4. CSMBS - 5 Commercial health insurance - 6. Elderly - 7. Disabled - 8. Other...................................... | | | | | | | | | | | | | 14 - 21 | | |
| **Module 2: Outcome (patients)** | | | | | | | | | | | | | | |  |
| 8. In the last three months, how the patients doing? | | | | | | | 9. what do the patients expect? | | | | | | |  |  |
| - - - 1 better     - 2 stable     - 4 worse     - 5 not sure/unknown | | | | | | | - - - 1 better     - 2 stable     - 4 worse     - 5 not sure/unknown | | | | | | | 22 - 29 |  |
| **10-11 for caregivers only** (if not, go to module 3) | | | | | | | | | | | | | |  |  |
| 10. As a caregiver, what you do think of patient’s life nowadays? (Can select more than one) | | | | | | | | | | | | | | 30 - 33 |  |
| - 1 more hopeful   - - 2 more physical tiring | | | | | | - - - 3 less hopefull     - 4 less freedom/autonomy | | | | | | | |  |  |
| 11. What do the patients need to get a better life? (Can select more than one) | | | | | | | | | | | | | | 34 - 43 |  |
| - 1 more family income   - 2 more family wealth   - 3 more caregivers   - 4 less family expenditures   - 5 more closely care by doctors and nurses   - 6 more understanding from others     - 7 more knowledge of caregiver     - 8 more channel to see a doctor     - 9 other ............................................................... | | | | | | | | | | | | | |  |  |

| **Module 4 Assessment criteria** | | | |  |
| --- | --- | --- | --- | --- |
| **4.1 Accessibility** | **NO** | **YES** | **N/A** | **CODE** |
| 1. Can FCT be reached all the time? |  |  |  | 77 |
| 1. Does hospital has an extended OPD? |  |  |  | 78 |
| 1. Does the patients get the care on the same day of arrival? |  |  |  | 79 |
| 1. Is it convenient at the regular care? |  |  |  | 80 |
| **Does FCT recommend these issues? (Can select more than one)** | **NO** | **YES** | **N/A** | **CODE** |
| 1. Healthy food |  |  |  | 81 |
| 1. Physical exercise |  |  |  | 82 |
| 1. Weight control |  |  |  | 83 |
| 1. Smoking |  |  |  | 84 |
| 1. Alcohol drinking |  |  |  | 85 |
| 1. Handwashing |  |  |  | 86 |
| 1. Medication |  |  |  | 87 |
| 1. Health concerns |  |  |  | 88 |
| 1. Fall prevention |  |  |  | 89 |
| 1. Osteoporosis |  |  |  | 90 |
| 1. Self-care of menopause |  |  |  | 91 |
| 1. Cancer screening |  |  |  | 92 |
| 1. Other…. |  |  |  | 93 –94 |
| **In the past year, did the patient receive these services ?** | **NO** | **YES** | **N/A** | **CODE** |
| 1. Influenzas vaccine |  |  |  | 95 |
| 1. Measuring blood sugar |  |  |  | 96 |
| 1. Measuring blood pressure |  |  |  | 97 |
| 1. Measuring serum lipid |  |  |  | 98 |
| 1. Weight measure |  |  |  | 99 |
| 1. Other............................... |  |  |  | 100-101 |
| **4.2 Disease management** | **NO** | **YES** | **N/A** | **CODE** |
|  |  |  |  |  |
| 1. Can patient request to see their medical history record when needed? |  |  |  | 102 |
| 1. Can patient obtain a copy of their exam finding? |  |  |  | 103 |
| **Patient experiences** | **NO** | **YES** | **N/A** | **CODE** |
| 1. Did patients experience miscommunication among care team that make patients confused? |  |  |  | 104 |
| 1. If referred, is there an advanced appointment for their referral visit?   (If have not been referred, move to 4.3) |  |  |  | 105 |

| **4.2.3 Questions regarding the consultations to specialists**  **(If not been consulted, move to 4.3)** | **NO** | **YES** | **N/A** | **CODE** |
| --- | --- | --- | --- | --- |
| 1. If referred, does primary care physicians know? |  |  |  | 106 |
| 1. Does primary care physicians ask about what happened when referred? |  |  |  | 107 |

| **4.3 People-centerness** | **NO** | **YES** | **N/A** | **CODE** |
| --- | --- | --- | --- | --- |
| **4.3.1 Continuous care** |  |  |  |  |
| 1. Spend time to relive patient’s anxiety |  |  |  | 108 |
| 1. Respect concerns of patients |  |  |  | 109 |
| 1. Know patients well |  |  |  | 110 |
| 1. Answer and explain patients well |  |  |  | 111 |
| 1. Identify and clarify pros and cons of treatment options |  |  |  | 112 |
| 1. Give phone number to patients can consult when having problems |  |  |  | 113 |
|  | **NO** | **YES** | **N/A** | **CODE** |
| 1. Do hospital staffs ask for permission of treatment from family members? |  |  |  | 114 |
| 1. Does physician visit patient’s home? |  |  |  | 115 |
| 1. Does patient recommend hospital to family member? |  |  |  | 116 |
| **4.4 Support of self-care**  **(if no chronic illness, please skip)** | **NO** | **YES** | **N/A** | **CODE** |
| **4.4.1 Understanding of clinical management plans** |  |  |  |  |
| 1. Have FCT informed the cause of disease and how to deal with it? |  |  |  | 117 |
| 1. Have FCt informed the methods of treatment? |  |  |  | 118 |
| 1. Is the patient confident to take care of oneself at home? |  |  |  | 119 |
| 1. Have FCT informed how to prevent complications? |  |  |  | 120 |
| 1. Have FCT made an appointment for the next visits? |  |  |  | 121 |
| 1. Have FCT visited patient’s home? |  |  |  | 122 |
| 1. Does FCT advise on healthy diets or physical exercise |  |  |  | 123 |

- Thank you, and say good bye!
