# Supplementary material for: Assessing system-based trainings for primary care teams and quality-of-life of patients with multimorbidity in Thailand: patient and provider surveys
Source: BMC Fam Pract. 2019 Jun 17;20:85. doi: 10.1186/s12875-019-0951-6 (PMC6580542; doi:10.1186/s12875-019-0951-6)
Supplement: Supplementary file 2 — Semi-structured Interview Guide for the Leaders of Family Care Team. (DOCX 26 kb) [file 12875_2019_951_MOESM2_ESM.docx]

**Additional file 1**

**Semi-structure Interview Guide for the Leaders of Family Care Team**

1. Policy communication, attitude and policy deployment

1.1 Would you please let us know how you implement the policy, and who do you report to? (Probe)

- Any delegation?
- Any meeting/orientation?
- Any training/capacity building?
  1. How did you feel when learning about this policy? (Probe)
- Is it a new way of working?
- Is it similar or overlapping with the existing work process? How so?
- What is the attitude of your colleagues?

1. Process of team set up

2.1 What was your team building process? (Probe)

- How many people in the catchment? How many families? How many teams did you set up?
- Who are the team members?
- How do you prepare your team? Any crucial communication?

1. Structure and Organization
   1. Please share us your success and the process you have used for building a multi-disciplinary team? (Probe)

- Problems/obstacles?
- Critical success factors?
- Level of commitment/collaboration?
  1. How do team members determine the methods for communication with each other? Any obstacles? (Probe)
  2. How do you set up management process? How to select the team leaders? (Probe)
- How to select team leaders?
- Criteria? Characteristics of team members?
- How to determine team members’ responsibilities?
- Any new regulations set up for FCT?
- Any collaborations with other local authorities?

1. Action/ activities (Probe):

- What activities has been started? How to prioritize them?
- Acceptance from the targeted population/people?
- How many areas of implementation? How to prioritize them?
- Interesting experiences of activities with teams in local areas?
- Any preparation of the family members? How do you work with team?
- Problems, challenges, success?
- Feedback from team members?
- How do you use manuals or follow clinical guidelines?
- Before action review (BAR) and after action review (AAR) of the home visits?

1. Results so far/measure?
   1. Any change of population that has been served? (e.g. elderly, disabilities, end-of-life patients? Generational population?)
   2. Response from patients, relatives? Collaboration? Changes?
   3. Measures of satisfactions and health outcomes?
   4. Changes of providers’ attitude and/or working process?
2. What are the key success factors of such FCT activities?
3. Any other recommendation for other FCTs and policy makers?
4. Any demands for supports to continue implementing effective FCT?
