# Supplementary material for: Assessing system-based trainings for primary care teams and quality-of-life of patients with multimorbidity in Thailand: patient and provider surveys
Source: BMC Fam Pract. 2019 Jun 17;20:85. doi: 10.1186/s12875-019-0951-6 (PMC6580542; doi:10.1186/s12875-019-0951-6)
Supplement: Supplementary file 3 — Questions for the Members of Family Care Team. (DOCX 2397 kb) [file 12875_2019_951_MOESM3_ESM.docx]

**Additional file 2**

**Questions for the Members of Family Care Team**

| Variable | Sequence | | | Region | | | | | | | | | | | | | Province | | | | District |
| --- | --- | --- | --- | --- | --- | --- | --- | --- | --- | --- | --- | --- | --- | --- | --- | --- | --- | --- | --- | --- | --- |
| CODE | 1 | | | 2 | | | | | | | | | | | | | 3 | | | | 4 |
| **Module 1 FCT** | | | | | | | | | | | | | | | | | | | | | CODE |
| 1. Which team are you in? | | | | | | | | | | | | | | | | | | | | | 5 |
| - 1 District team | | | | - 2 Subdistrict team | | | | | | | | | | | | | - 3 Village team | | | |  |
| 1. Your position/profession | | | | | | | | | | | | | | | | | | | | | 6-7 |
| - 2.1 Physician - 2.2 Dentist - 2.3 Pharmacist - 2.4 Nurse - 2.5 Psychologist - 2.6 Physiotherapist | | | | - 2.7 Researcher/Officer - 2.8 Dental auxiliary/nurse - 2.9 Folk doctor - 2.10 Nutritionist/dietitian - 2.11 Other health personnel - 2.12 Community health volunteer | | | | | | | | | | | | | - 2.13 Local government - 2.14 Chief of subdistrict - 2.15 Chief of village - 2.16 Leader - 2.17 Other volunteer - 2.18 other.................... | | | |  |
| 1. At the district level (community hospital), who are FCT member? (Can select more than one) | | | | | | | | | | | | | | | | | | | | | 8-26 |
| - 3.1 Physician - 3.2 Dentist - 3.3 Pharmacist - 3.4 Nurse - 3.5 Psychologist - 3.6 Physiotherapist | | | | - 3.7 Researcher/Officer - 3.8 Dental auxiliary/nurse - 3.9 Folk doctor - 3.10 Nutritionist/dietitian - 3.11 Other health personnel - 3.12 Community health volunteer | | | | | | | | | | | | | - 3.13 Local government - 3.14 Chief of subdistrict - 3.15 Chief of village - 3.16 Leader - 3.17 Other volunteer - 3.18 other.................... | | | |  |
| 4. What is the process of your FCT to contextualize your work? (Can select more than one) | | | | | | | | | | | | | | | | | | | | | 27-31 |
| - 4.1 Improve the work process from Context Based Learning e.g. geography, culture, lifestyles - 4.2 Knowledge Management - 4.3 other...................................... - 4.4 None/don’t know/not sure | | | | | | | | | | | | | | | | | | | | |  |
| 5. What are channel of communication between team members? (Can select more than one) | | | | | | | | | | | | | | | | | | | | | 32-40 |
|  | | |  | | | | | | | | | | | | |  | | | | |  |
| - 5.1 Mobile phone - 5.2 Office phone - 5.3 Fax - 5.4 Phone | | | | | | | | | - 5.5 Facebook - 5.6 SMS (Line) - 5.7 Email - 5.8 Other...................................... | | | | | | | | | | | |  |
| 6. What are incentives from your organization? (Can select more than one) | | | | | | | | | | | | | | | | | | | | | 41-49 |
| - 6.1 Budget - 6.2 Reward/promotion - 6.3 Gift - 6.4 Award | | | | | | | - 6.5 Additional manpower - 6.6 Closely monitoring/supervising - 6.7 Encouragement - 6.8 Other...................................... | | | | | | | | | | | | | |  |
| 7. What is your feeling to FCT? | | | | | | | | | | | | | | | | | | | | | 50 |
| - 1 Proud to be team member | | | | | | |  | - 2 Indifferent | | | | | | - 3 Not sure | | | | | | 4 Not proud |  |
| 8. Regular meetings at the district level? | | | | | | | | | | | | | | | | | | | | | 51 |
| - 1 None | | | - 2 yes | | | | | | | | | | | | | - 3 Not sure/unknown | | | | |  |
| 9. When was the last team meeting at the district level?................ | | | | | | | | | | | | | | | | | | | | | 52 |
| **Mode 2 Roles** | | | | | | | | | | | | | | | | | | | | | **CODE** |
| 10. What is your role? | | | | | | | | | | | | | | | | | | | | | 53 |
| - - - 1 Team leader | | - - - 2 Team member | | | | | | | | | - - - 3 Consultant | | | | | | | | - - - 4 Volunteer | |  |
| 11. Gender | | | | | | | | - - - 1 male | | | | | | | | | | - - - 2 female | | | 54 |
| 12. age | | | | | | | | ………………….yr | | | | | | | | | | | | | 55 |
| 13. work duration | | | | | | | | ………………….yr | | | | | | | | | | | | | 56 |
| 14. Months of joining FCT ………………….mo | | | | | | | | | | | | | | | | | | | | | 57 |
| **Module 3 : Attitude** | | | | | | | | | | | | | | | | | | | | | CODE |
| 15. How much your patients benefits from FCT? | | | | | | | | | | | | | | | | | | | | | 58 |
| - 1 Maximal - 2 A lot | | | | | - 3 Moderate - 4 Little | | | | | | | | | | - 5 Minimal - 6 Not sure | | | | | |  |
| 16. Is the teamwork between community hospitals and subdistrict health centers help taking care of NCDs patients? | | | | | | | | | | | | | | | | | | | | | 59 |
| - 1 No | | | | | - 2 Yes | | | | | | | | | | - 3 Not sure | | | | | |  |
| 17. Is FCT helping family and community more accessible to healthcare? | | | | | | | | | | | | | | | | | | | | | 60 |
| - 1 No | | | | | - 2 Yes | | | | | | | | | | - 3 Not sure | | | | | |  |
| 18. Is FCT helping family and community more quality of life? | | | | | | | | | | | | | | | | | | | | | 61 |
| - 1 No | | | | | - 2 Yes | | | | | | | | | | - 3 Not sure | | | | | |  |
| 19. Is FCT helping family and community reduce the health risks? | | | | | | | | | | | | | | | | | | | | | 62 |
| - 1 No | | | | | - 2 Yes | | | | | | | | | | - 3 Not sure | | | | | |  |
| 20. Is FCT considered a good policy and should be continued? | | | | | | | | | | | | | | | | | | | | | 63 |
| - 1 No | | | | | | - 2 Yes | | | | | | | - 3 Not sure | | | | | | | |  |
| 21. Is FCT considered a change of wok process towards more teamwork? | | | | | | | | | | | | | | | | | | | | | 64 |
| - 1 No | | | | | | - 2 Yes | | | | | | | - 3 Not sure | | | | | | | |  |
| 22. Is FCT more involving family and community in the care planning? | | | | | | | | | | | | | | | | | | | | | 65 |
| - 1 No | | | | | - 2 Yes | | | | | | | | | | - 3 Not sure | | | | | |  |
| 23. Are your patients receiving a comprehensive care including care in the referral systems? | | | | | | | | | | | | | | | | | | | | | 66 |
| - 1 No | | | | | | - 2 Yes | | | | | | | - 3 Not sure | | | | | | | |  |
| 24. Suggestions (Can select more than one)  🗌 1 Regular team meetings  🗌 2 Find more physicians to be successors of the team leaders  🗌 3 Leaders led the home visits | | | | | | | | | | | | | | | | | | | | | 67-69 |
| **Module 4 : Training, knowledge, confidence** | | | | | | | | | | | | | | | | | | | | | CODE |
| 25. What kinds of training did you received? (Can select more than one) | | | | | | | | | | | | | | | | | | | | | 70-81 |
| - 25.1 DHS - 25.2 District Health System (DHS) Appreciation - 25.3 Supervisors in primary care practice - 25.4 CUP leadership - 25.5 Community Physiotherapist Learning - 25.6 Community Pharmacist Learning | | | | | | | | | | - 25.7 District Health Management Learning (DHML) - 25.8 Community Pharmacy in primary care - 25.9 Training of public health workers in primary care - 25.10 CPG - 25.11 other ................................................... | | | | | | | | | | |  |
| 26. How much your knowledge of FCT? | | | | | | | | | | | | | | | | | | | | | 82 |
| - 1 None - 2 Fair | | | | | | | | | | - 3 Good - 4 Expert | | | | | | | | | | |  |
| 27. How much confidence you have in working as a FCT member? | | | | | | | | | | | | | | | | | | | | | 83 |
| - 1 Totally not confident - 2 Not very confident | | | | | | | | | | - 3 Confident - 4 Very confident | | | | | | | | | | |  |
| 28. What are services your provide? (Can select more than one) | | | | | | | | | | | | | | | | | | | | | 84-94 |
| - 28.1 Health promotion - 28.2 Long-term care - 28.3 Counselling - 28.4 Coordination - 28.5 Palliative care | | | | | | | | | | - 28.6 Medcial treatment - 28.7 Rehabilitation - 28.8 Write statistical reports - 28.9 Training of local government - 28.10 other………………………………… | | | | | | | | | | |  |
| 28. What are your major roles in FCT? (Can select more than one) | | | | | | | | | | | | | | | | | | | | | 95-103 |
| - 29.1 Care provider - 29.2 Counsellor - 29.3 Problem solver - 29.4 Learning leader | | | | | | | | | | - 29.5 Coordinator - 29.6 Surveillance officer - 29.7 Role model - 29.8 Other....................................... | | | | | | | | | | |  |
| 30. What kind of training do you want?  🗌 1. Family practice  🗌 2. Data analysis  🗌 3. Process facilitator  🗌 4. Other............................................................................ | | | | | | | | | | | | | | | | | | | | | 104 |
| 31. What is the process in your organization to support and supervise FCT?  (Can select more than one) | | | | | | | | | | | | | | | | | | | | | 105-115 |
| - 31.1 Creating standards - 31.2 Creating CPGs - 31.3 Design the records, notes, memos - 31.4 Additional budget - 31.5 Additional manpower | | | | | | | | | | | | - 31.6 Additional training - 31.7 Supervising - 31.8 Reform of organizational structure - 31.9 Consultation and referral systems - 31.10 other........................................ | | | | | | | | |  |
| **Module 5: Information systems** (Only the district ad subdistrict levels) | | | | | | | | | | | | | | | | | | | | |  |
|  | | | | | | | | | | | | | | | | | | | | |  |

| Patient groups | 32. Is there a specific database for patients? | | | | | 33. is there a continuously improvement of information systems? | | | |  | |
| --- | --- | --- | --- | --- | --- | --- | --- | --- | --- | --- | --- |
|  | No | | Yes | | Don’t know | No | Yes | | Don’t know |  |  |
| 1. Disabled |  | |  | |  |  |  | |  | CODE 32  116-125 | |
| 1. Diabetes |  | |  | |  |  |  | |  |  |  |
| 1. HT |  | |  | |  |  |  | |  |  |  |
| 1. Elderly |  | |  | |  |  |  | |  |  |  |
| 1. HIV/AIDS |  | |  | |  |  |  | |  |  |  |
| 1. Asthma |  | |  | |  |  |  | |  | CODE  33  126-135 | |
| 1. COPD |  | |  | |  |  |  | |  |  |  |
| 1. Mental illness |  | |  | |  |  |  | |  |  |  |
| 1. Other............... |  | |  | |  |  |  | |  |  |  |
| 34. Have you seen data analyses of these patients and used them for quality improvement? | | | | | | | | | | CODE |  |
| Patient groups | | None | | Yes (last time was…months ago) | | | | Not sure/unknow | |  |  |
| 1. พิการ | |  | |  | | | |  | | CODE  34  136-154 |  |
| Disabled | |  | |  | | | |  | |  |  |
| Diabetes | |  | |  | | | |  | |  |  |
| HT | |  | |  | | | |  | |  |  |
| Elderly | |  | |  | | | |  | |  |  |
| HIV/AIDS | |  | |  | | | |  | |  |  |
| Asthma | |  | |  | | | |  | |  |  |
| COPD | |  | |  | | | |  | |  |  |
| Mental illness | |  | |  | | | |  | |  |  |

**Thank you!**
